# Impact of transferrin levels on iron accumulation in transfusion-dependent beta-thalassemia: A genotype-specific analysis

**DOI:** 10.5937/jomb0-54839

**Published:** 2025-10-28

**Authors:** Yidan Liang, Xinhua Zhang, Binbin Huang, Yushan Huang, Liuhua Liao, Yueyan Huang, Ken Huang, Jinquan Lao, Xiaoqin Feng, Bin Lin, Xingjiang Long, Zhixiang Liu, Weijian Zhu, Lian Yu, Deguo Tang, Tianyu Zhong, Yuhua Ye, Xiangmin Xu

**Affiliations:** 1 Innovation Center for Diagnostics and Treatment of Thalassemia, Nanfang Hospital, Southern Medical University, Guangzhou 510515, Guangdong, China; 2 Department of Medical Genetics, School of Basic Medical Sciences, Southern Medical University, Guangzhou 510515, Guangdong, China + Guangdong Genetics Testing Engineering Research Center, Guangzhou 510515, Guangdong, China; 3 Department of Hematology, 923(rd) Hospital of the People's Liberation Army, Nanning, Guangxi 530021, China; 4 Department 1 of Internal Medicine, Sixth People's Hospital of Nanning, Nanning, Guangxi 530022, China; 5 College of Life Sciences, University of Chinese Academy of Sciences, Beijing 100049, China 7BGI-Shenzhen, Shenzhen 518083, China; 6 Department of Pediatrics, Huizhou Central People's Hospital, Huizhou, Guangdong 516001, China; 7 Department of Pediatrics, Affiliated Hospital of Youjiang Medical University for Nationalities, Baise, Guangxi 533000, China; 8 Department of Pediatrics, Liuzhou Worker's Hospital, Liuzhou, Guangxi 545005, China; 9 Department of Pediatrics, Nanfang Hospital, Southern Medical University, Guangzhou 510515, Guangdong, China; 10 Guangzhou Huayin Healthcare Group Co., Ltd., Guangzhou 510663, Guangdong, China; 11 Guangzhou Jiexu Gene Technology Co., Ltd., Guangzhou 510530, Guangdong, China; 12 Department of Pediatrics, Liuzhou People's Hospital, Liuzhou, Guangxi 545001, China; 13 Department of Medical Genetics, Maternal and Child Health Hospital, Heyuan 517000, China; 14 Department of Hematology, Zhuhai People's Hospital (Zhuhai Hospital Affiliated with Jinan University), Guangdong Zhuhai 519000, China; 15 Department of Hematology, Longyan First Hospital Affiliated to Fujian Medical University, Longyan 364000, China; 16 Maternal and Child Health Hospital of Yongzhou City, Yongzhou, Hunan 425000, China; 17 Department of Laboratory Medicine, First Affiliated Hospital of Gannan Medical University, Ganzhou 341000, Jiangxi, China

**Keywords:** transferrin, serum ferritin, transfusion-dependent beta-thalassemia, iron overload, transferin, serumski feritin, transfuziono zavisna beta-talasemija, preopterećenje gvožđem

## Abstract

**Background:**

Serum ferritin (SF) monitors secondary iron overload in beta-thalassemia (b-thalassemia). Transferrin (TRF) has been shown to reverse iron accumulation in experimental models, but its role in transfusion-dependent beta-thalassemia (TDT) patients remains unclear. This study aims to explore the relationship between TRF and SF in TDT patients and to reveal the unique connection between specific genotypes and iron metabolism, providing potential therapeutic targets for clinical practice.

**Methods:**

This cross-sectional study includes 817 TDT patients (b0/b0 genotype: n=560; b0/b+ genotype: n=257). We use genotype-phenotype analysis and employ logistic regression and restricted cubic spline (RCS) curves to assess the association between TRF and SF.

**Results:**

Significant differences were observed between the b0/b0 and b0/b+ genotypes in terms of age at first transfusion, transfusion requirements, chelation initiation age, reticulocyte count, red blood cell count, red cell distribution width-coefficient of variation (RDW-CV), fetal haemoglobin (HbF) level, splenomegaly, and SF. b0/b0 patients presented with more severe clinical phenotypes. SF was significantly associated with TRF, HbF, RDW-CV, and chelation therapy. RCS analysis revealed a dose-response relationship with a negative linear correlation between TRF and SF (OR=0.26, P&lt;0.001), indicating that higher TRF levels are linked to lower SF risk.

**Conclusions:**

This study systematically confirms for the first time a significant negative correlation between high TRF levels and high SF risk in TDT patients. This new finding may help clinicians more effectively manage iron overload, especially in patients with different genotypes.

## Abstract (sr)

**Uvod:**

Serumski feritin (SF) se koristi za praćenje sekundarnog preopterećenja gvožđem kod beta-talasemije (b-talasemija). Pokazano je da transferin (TRF) može da preokrene nakupljanje gvožđa u eksperimentalnim modelima, ali njegova uloga kod pacijenata sa transfuziono zavisnom beta-talasemijom (TDT) ostaje nejasna. Cilj ove studije je da istraži odnos između TRF-a i SF-a kod TDT pacijenata i da otkrije jedinstvenu povezanost između specifičnih genotipova i metabolizma gvožđa, pružajući potencijalne terapijske mete za kliničku praksu.

**Metode:**

Ova studija preseka obuhvata 817 pacijenata sa TDT-om (genotip b0/b0: n=560; genotip b0/b+: n=257). Primenjena je analiza genotip-fenotip, logistička regresija i krive sa ograničenim kubnim splinom (RCS) kako bi se procenila povezanost između TRF-a i SF-a.

**Rezultati:**

Primećene su značajne razlike između genotipova b0/b0 i b0/b+ u pogledu starosti pri prvoj transfuziji, potreba za transfuzijom, starosti započinjanja helacione terapije, broja retikulocita, broja crvenih krvnih zrnaca, širine distribucije crvenih krvnih zrnaca-koeficijenta varijacije (RDW-CV), nivoa fetalnog hemoglobina (HbF), splenomegalije i SF-a. Pacijenti sa b0/b0 genotipom imali su teže kliničke fenotipove. SF je bio značajno povezan sa TRF-om, HbF-om, RDW-CV-om i helacionom terapijom. RCS analiza je pokazala odnos doze i odgovora sa negativnom linearnom korelacijom između TRF-a i SF-a (OR=0,26, P&lt;0,001), što ukazuje da su viši nivoi TRF-a povezani sa nižim rizikom od visokog SF-a.

**Zaključak:**

Ova studija po prvi put sistematski potvrđuje značajnu negativnu korelaciju između visokih nivoa TRF-a i visokog rizika od povećanog SF-a kod TDT pacijenata. Ovo novo otkriće može da pomogne kliničarima da efikasnije upravljaju preopterećenjem gvožđem, posebno kod pacijenata sa različitim genotipovima.

## Introduction

Beta-thalassemia (β-thalassemia) is a common monogenic hereditary disorder caused by mutationsin the β-globin gene, affecting approximately 1% to 5% of the global population [1]. These mutations result in either reduced synthesis (β^+^ type) or complete absence (β^0^ type) of the β-globin chains, leading to a spectrum of clinical manifestations ranging from mild to severe [1] [2], including hepato splenomegaly, growth retardation, and skeletal deformities. β-thalassemia is categorised into non-transfusion-dependent beta-thalassemia (NTDT) and transfusiondependent beta-thalassemia (TDT) types based on the severity of clinical symptoms [3].

Patients with TDT require regular blood transfusions to suppress the accelerated apoptosis of erythroid precursors. While this essential treatment is life-sustaining, it leads to long-term iron overload [4] [5]. Increased intestinal iron absorption and secondary iron overload [6] can severely compromise patients’ health. Transferrin (TRF), the primary iron transport protein in plasma, is critical in regulating iron metabolism [7]. In β-thalassemia research, TRF has been shown to modulate iron metabolism by inducing hepcidin expression [8], demonstrating its potential toreduce iron accumulation in β-thalassemia mouse models [9] [10].

Serum ferritin (SF), another key biochemical marker, is commonly used to monitor iron overload in the body [11]. Changes in SF levels reflect fluctuations in iron storage and are closely linked to the long-term prognosis of patients [12] [13]. Elevated SF levels are typically associated with iron overload [14], particularly in TDT patients. Therefore, monitoring SF levels is crucial for assessing the effectiveness of iron chelation therapy [15]. In TDT patients, timely and effective management of iron overload is essential for preventing the development of complications such as heart disease, liver disorders, and other related conditions.

This study’s hypothesis is based on the observed phenomenon that patients with different β-thalassemia genotypes exhibit significant variations in iron overload. We propose that this difference may be partially attributed to the varying roles of TRF in iron metabolism. In specific genetic contexts, the expression and function of TRF may regulate iron absorption, utilisation, and storage through unknown mechanisms, thereby influencing the iron overload status of patients. In a large cohort study involving 817 TDT patients, we aim to analyse the interaction between TRF and iron overload markers, such as SF, to determine whether these interactions differ by genotype and assess their potential implications for disease management.

The discovery of this relationship may reveal new biomarkers or therapeutic targets that could be used to develop novel treatment strategies or optimise existing iron chelation therapies. For instance, if TRF is crucial in regulating iron absorption and storage, therapies targeting TRF might be more effective for patients with genotypes that result in higher TRF expression.

The primary objective of this study is to evaluate the specific role of TRF in managing iron overload and to understand how its dynamic relationship with SF influences iron metabolism. We plan to employ advanced biostatistical methods and bioinformatics tools to analyse clinical and biochemical data from patients, aiming to identify interaction patterns between TRF and SF and assess their potential impact on disease progression.

Additionally, by comparing data from patients with different genotypes, we hope to gain deeper insights into how the genetic background of iron metabolism affects treatment response. This analysis helps us understand why some patients respond poorly to standard therapies and may guide the shift toward more personalised treatment approaches.

## Materials and methods

### Experimental methods

### Study design and participant selection

Approved by the Ethics Committee of Nanfang Hospital, Southern Medical University (Approval No. NFEC-2019-039), this study adheres to the Declaration of Helsinki, with informed consentobtained from all patients. Conducted between June 2019 and June 2022 across five provinces in southern China (Guangxi, Guangdong, Fujian, Jiangxi, and Hunan), the research aimed to investigate the iron metabolism characteristics of patients with TDT. A total of 1,020 patients were initially recruited from local medical institutions. After applying strict exclusion criteria, such as age under 3 years, pregnancy, or the presence of active inflammatory diseases, 817 eligible patients were included in the study. These patients were further classified into 560 β^0^/β^0^ homozygotes and 257 β^0^/β^+^ compound heterozygotes, with their HBB genotypes confirmed.

### Genotype confirmation

The β-thalassemia genotypes of each participant were confirmed by collecting peripheral blood samples, with initial screening performed using reverse dot blot hybridisation (RDB) and gap-PCR. All samples were subsequently analysed using next-generation sequencing (NGS) technology to ensure the accuracy and reliability of the genotyping, allowing for a more detailed investigation of specific gene mutations and variations.

### Clinical data collection

Comprehensive clinical data were collected through detailed medical record reviews and face-to-face interviews. The included basic demographic information (age, gender, weight, height), transfusion-related details (age at first transfusion, transfusion frequency), and detailed records of chelation therapy. All data were meticulously entered into a specially designed electronic database to ensure accuracy and facilitate subsequent analysis.

### Physiological measurements

All patients underwent liver and spleen ultrasounds using high-resolution equipment provided byGE Healthcare. It allowed for a precise assessment of these key organs’ size and structural changes, which is crucial for monitoring disease progression and evaluating changes in iron overload.

### Laboratory tests

(1) Hemoglobin HbA, HbA_2_, and HbF Measurement: Approximately 2 mL of venous blood was collected from each patient into an EDTA anticoagulation tube for haemoglobin analysis. The HbA, HbA_2_, and HbF levels were measured using high-performance liquid chromatography (HPLC) with the Bio-Rad Variant II system, following the manufacturer’s instructions. This method enables precise quantification of different haemoglobin subtypes, which is crucial for assessing haemoglobin synthesis and iron utilisation efficiency [16].

(2) Complete Blood Count: A full blood count was conducted using the Sysmex pocH-100i automated haematology analyser, providing data for a comprehensive assessment of the patient’s overall blood health.

(3) Iron Metabolism-Related Measurements: SF was measured using a chemiluminescent immunoassay on Siemens’ Dade Behring BN II system. Serum iron (SI) and total iron-binding capacity (TIBC) were determined using Leadman’s TIBC microporous method kit. Transferrin saturation (TS) was then calculated based on these values to evaluate iron metabolism comprehensively.

### Data processing and analysis

### Normality testing and inter-group comparison methods

At the initial stage of analysis, all variables underwent a normality test. For variables that did not follow a normal distribution, the Mann-Whitney U test was used to compare the two groups. Categorical variables were compared using the X^2^ test. The significance level for all statistical inferences was set at *p*<0.05.

### Criteria for iron overload classification

According to the *Chinese guidelines for diagnosing and treating TDT (Subspecialty Groups of Hematology, 2018)* and related clinical observations, SF levels exceeding 2,500 ng/mL indicate severe iron overload. Based on this, SF levels were further categorised into 2,500 ng/mL and >2,500 ng/mL to investigate the impact of different iron overload states.

### Core analysis of the relationship between TRF and SF

A multivariate logistic regression model analysed the relationship between TRF and SF levels. Initially, univariate logistic regression was employed to identify variables significantly associated with SF levels. All variables with p<0.1 were included in the multivariate model to explore potential associations between TRF and SF, with selection performed using a stepwise backward regression approach. The final model reported the odds ratios (ORs) and 95% confidence intervals (CIs) for each factor, directly reflecting the validation of the core research hypothesis.

### Sensitivity analysis

A sensitivity analysis was conducted on the core results, with clinical stratification based on patients’ HBB genotype, pre-transfusion Hb levels, and liver and spleen size. This analysis aimed to assess the stability and consistency of the relationship between SF and TRF across different clinical subgroups.

### Dose-response quantitative analysis

The dose-response relationship between TRF levels and the risk of elevated SF was further quantified using the restricted cubic spline (RCS) method. This analysis adjusted for all clinically and biologically relevant variables, offering a progressively detailed analysis from basic to more complex models.

### Statistical tools and techniques

All basic descriptive and inferential statistical analyses were performed using SPSS software (version 23.0). For more complex graphical presentations and data analysis, the R language’s ggplot2 package (version 4.2.1) was employed to ensure the results’ accuracy and the charts’ professional quality.

## Results

### Genotype-phenotype analysis in β-thalassemia patients

We analysed clinical, haematological, iron-related biochemical, and abdominal ultrasound data from 817 patients, as summarised in Table 1. The median age of the patients was 10 years, with 43% female. Patients were classified as either β^0^/β^0^ or β^0^/β^+^ to examine the impact of genotype on disease severity. Patients in the b0/b0 group showed an earlier initiation of transfusion (*p*<0.001) and a higher transfusion demand (*p*<0.001) aimed at suppressing ineffective erythropoiesis (0.02×10^12^/L vs 0.06×10^12^/L, p<0.001), which in turn led to an earlier start of chelation therapy (*p*<0.001). Hematologically, the β^0^/β^0^ group had lower red blood cell (RBC) counts, lower red cell distribution width (RDW), and lower fetal haemoglobin (HbF) levels, with splenomegaly being more common. These findings likely illustrate the influence of long-term transfusion in ameliorating anaemia, progressively diminishing red blood cell size heterogeneity [17]. Regarding iron metabolism, the majority (758/817, 92.7%) of patients suffered from iron overload, with 61.3% (465/758) having SF levels exceeding 2,500 ng/mL. Overall, 61.9% (347/560) of patients in the b0/b0 group and 46.7% (120/257) of patients in the β^0^/β^+^ group had SF levels exceeding 2,500 ng/mL, with significantly higher SF levels observed in the b0/b0 group (p<0.001). The β^0^/β^0^ patients comprised 68.5% (560/817) of the cohort. The median TRF level was 1.36 g/L, below the normal range (2.0–3.6 g/L), with only 11 patients within the normal range. However, no significant differences in TS or TRF levels were observed between the β^0^/β^0^ and β^0^/β^+^ groups.

**Table 1:** Baseline of characteristics of patients with β-thalassemia stratified by HBB genotype. Note: Abbreviations: Hb, haemoglobin; RBC, red blood cell count; RDW-CV%, red cell distribution width-coefficient of variation; Hct, hematocrit; Ret, reticulocytes; HbF, fetal haemoglobin; SF, serum ferritin; TS, transferrin saturation; TRF, serum transferrin. Data represent median (interquartile range, IQR). †HBB genotype categories were defined as follows: (β^0^) codons 41-42-TTCT (37.1%), codons 17A>T (20.6%), IVS-II-654 C>T (16.4%), codons 71-72+A (3.6%), codons 54-58-TATGGGCAACCCT (0.12%), IVS-I-1 G>T (1.6%), codons 27/28+C (1.3%), codons 14-15+G (0.24%), codons 43G>T (0.61%), codons 95+A (0.06%), codons 37G>A (0.18%), codons 30A>G (0.06%), IVS-II-1 G>A (0.06%), Initiation codon ATG>AGG (0.06%); (β^+^), −28 A>G (11.6%), codons 26 GAG>AAG (3.9%), −29 A>G (1.1%), −90 C>T (0.12%), bnt1587 A>G (0.06%), IVS-II-5 G>C (0.36%), and Hb-Lepore (0.12%). Genotypes classified as homozygous β^0^ (β^0^/β^0^), and compound heterozygous b0 and β^+^ (β^0^/β^+^). ‡Annual blood transfusion volume was calculated as: blood transfusion frequency × blood transfusion units × 200 (mL)/body weight (kg). The data and percentages for each genotype group (β^0^/β^0^+ and β^0^/β) are calculated based on the number of patients within each respective group, not the entire patient population.

Clinical and phenotypic indices	Total (n=817)	β^0^/β^0^ (n=560)†	β^0^/β^+^ (n=257)†	*p*-value
Female, n (%)	352 (43.0)	234 (66.5)	118 (33.5)	0.27
Age (y)	10.1 (7.13–13.0)	9.8 (7.0–13.0)	11.0 (8.0–14.0)	0.003
High (cm)	130.0 (115.0–145.0)	128.5 (114.0–143.0)	133.0 (118.0–148.4)	0.004
Weight (kg)	26.0 (19.5–36.0)	25.3 (19.0–35.5)	27.6 (20.5–37.4)	0.04
Age at first transfusion<br>(months)	8.0 (5.0–18.0)	6.0 (4.0–10.0)	12.0 (7.0–36.0)	<0.001
Chelation-initiation age (yr)	4.0 (2.5–6.0)	3.0 (2.0–5.0)	5.0 (3.0–6.2)	<0.001
Annual blood transfusions<br>volume (mL/kg/yr) ‡	333.3 (266.7–402.7)	342.8 (274.2–411.1)	313.0 (251.1–376.1)	<0.001
Haematological parameters				
Hb (g/L)	92.0 (83.0–102.0)	92.0 (83.0–102.0)	93.0 (84.0–102.0)	0.48
RBC (10^12^/L)	3.4 (3.1–3.8)	3.3 (2.9–3.7)	3.6 (3.3–3.9)	<0.001
RDW-CV (%)	15.8 (13.7–20.5)	14.8 (13.5–18.4)	19.7 (14.8–25.1)	<0.001
Hct (%)	28.0 (25.2–31.0)	27.7 (24.9–31.0)	28.4 (26.1–31.1)	0.06
Ret (10^12^/L)	0.03 (0.02–0.06)	0.02 (0.02–0.04)	0.06 (0.03–0.13)	<0.001
HbF (g/L)	3.16 (1.71–6.36)	2.56 (1.42–5.18)	5.72 (2.73–11.72)	<0.001
Iron-related parameters				
SF (ng/mL)	2852.7 (1676.8–4495.0)	3060.9 (1947.1–4850.0)	2265.4 (1483.9–3412.4)	<0.001
TS (%)	75.7 (57.9–88.5)	76.3 (58.4–88.1)	73.8 (54.7–89.7)	0.29
TRF (g/L)	1.36 (1.20–1.53)	1.36 (1.19–1.53)	1.36 (1.21–1.53)	0.75
Ultrasonic imaging index, n (%)				
Hepatomegaly	473 (57.8)	323 (68.3)	150 (31.7)	0.85
Splenomegaly	429 (52.5)	268 (62.5)	161 (37.5)	<0.001
Splenectomy	81 (9.9)	50 (61.7)	31 (38.3)	0.16

The median duration of chelation therapy was 5.5 years. All patients required chelation therapy during the study period and adhered to daily medication as prescribed by their physicians. Regarding chelation regimens, 31.8% of patients used deferiprone, 32.8% were treated with deferasirox monotherapy, and 35.4% received a combination or alternation of two chelating agents.

### Logistic regression analysis of clinical factors affecting SF levels

**Figure 1:**
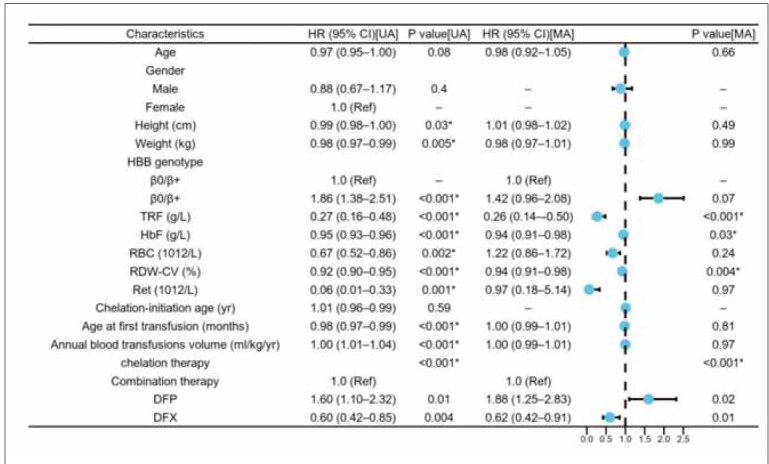
Correlation between relevant clinical factors and SF in logistic regression analysis.<br>Note: Hb, haemoglobin; OR, odds ratio; CI, confidence interval

The study used logistic regression analysis to explore the clinical factors influencing SF levels (Table 2, Figure 1). The multivariable-adjusted results showed that lower TRF levels were significantly associated with higher SF levels (OR=0.26, *p*<0.001), suggesting that TRF may play a key role in regulating iron overload. Other significant factors included HbF levels (OR=0.94, p=0.03), coefficient of variation of red cell distribution width (RDW-CV) (OR=0.94, *p*=0.004), and chelation therapy (p<0.001). However, genotype (β^0^/β^0^vs. β ^0^/β ^+^) was not significantly associated with an increased risk of elevated SF levels (OR=1.42, p=0.07). These findings suggest that TRF and HbF may have independent regulatory roles in iron overload management.

**Table 2:** Note: Abbreviations: RBC, red blood cell count; RDW-CV%, red cell distribution width-coefficient of variation; Ret, reticulocytes; HbF, fetal haemoglobin; TRF, serum transferrin; DFP, deferiprone; DFX, deferasirox; OR, odds ratio; CI, confidence interval. * represents a significant difference, *p*<0.05.

Variables	Univariate analysis		Multivariable analysis	
	OR (95% CI)	*p*-value	OR (95% CI)	*p*-value
Age	0.97 (0.95–1.00)	0.08	0.98 (0.92–1.05)	0.66
Gender				
Female	1.0 (Ref)			
Male	0.88 (0.67–1.17)	0.40	–	–
Height (cm)	0.99 (0.98–1.00)	0.03*	1.01 (0.98–1.02)	0.49
Weight (kg)	0.98 (0.97–0.99)	0.005*	0.98 (0.97–1.01)	0.99
HBB genotype				
β ^0^/β ^+^	1.0 (Ref)		1.0 (Ref)	
β ^0^/β ^0^	1.86 (1.38–2.51)	<0.001*	1.42 (0.96–2.08)	0.07
TRF (g/L)	0.27 (0.16–0.48)	<0.001*	0.26 (0.14–0.50)	<0.001*
HbF (g/L)	0.95 (0.93–0.96)	<0.001*	0.94 (0.91–0.98)	0.03*
RBC (10^12^/L)	0.67 (0.52–0.86)	0.002*	1.22 (0.86–1.72)	0.24
RDW-CV (%)	0.92 (0.90–0.95)	<0.001*	0.94 (0.91–0.98)	0.004*
Ret (10^12^/L)	0.06 (0.01–0.33)	0.001*	0.97 (0.18–5.14)	0.97
Chelation-initiation age (yr)	1.01 (0.96–0.99)	0.59	–	–
Age at first transfusion (months)	0.98 (0.97–0.99)	<0.001*	1.00 (0.99–1.01)	0.81
Annual blood transfusion volume<br>(mL/kg/yr)	1.00 (1.01–1.04)	<0.001*	1.00 (0.99–1.01)	0.97
Chelation therapy		<0.001*		<0.001*
Combination therapy	1.0 (Ref)		1.0 (Ref)	
DFP	1.60 (1.10–2.32)	0.01	1.88 (1.25–2.83)	0.02
DFX	0.60 (0.42–0.85)	0.004	0.62 (0.42–0.91)	0.01

### Stratified sensitivity analysis of the association between TRF and SF based on anaemia-related indicators

**Figure 2:**
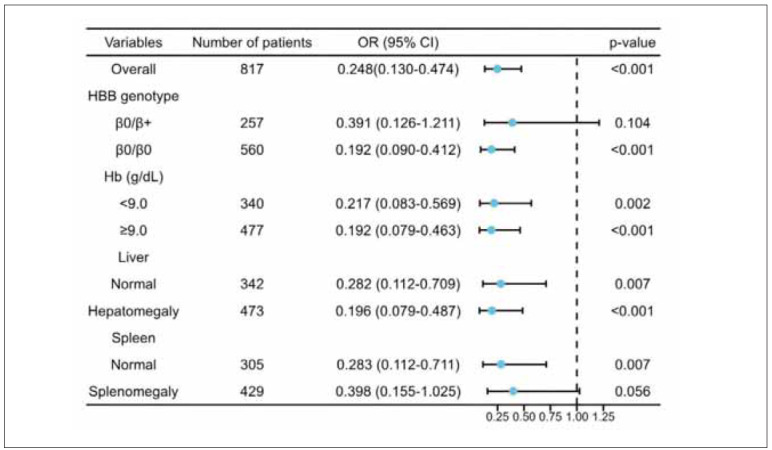
Forest plot of sensitivity analysis for the association between TRF and SF.<br>Note: The results indicate a strong correlation between TRF and SF

To assess the association between TRF and SF, we conducted a stratified analysis based on anaemiarelated indicators, including HBB genotype, Hb levels, and liver and spleen size. In these stratified models, the strength of the association between TRF and SF remained consistent with the primary model (Figure 2). The sensitivity analysis confirmed that the correlation between TRF and SF remained robust across various subgroups.

### Linear negative correlation between TRF and SF

**Figure 3:**
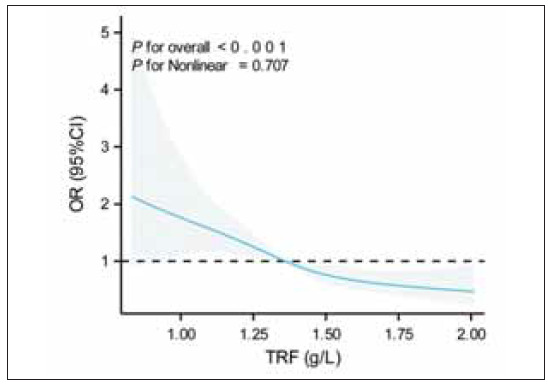
RCS curve analysis of TRF levels and pathological risk.<br>Note: The RCS curve analysis demonstrates the association between TRF and SF after adjusting for age, height, weight,HbF, RBC, RDW, Ret, HBB genotype, age at first transfusion, and annual transfusion volume. The results confirmed a negative linear correlation between TRF and SF. TRF, transferrin; OR, odds ratio; CI, confidence interval.

To further investigate the relationship between TRF and SF, patients were divided into quartiles (Q1-Q4) based on their TRF levels, and the association between TRF and SF was analysed (Table 3). The results indicated higher TRF levels (Q3 and Q4) were significantly associated with lower SF levels. Notably, in the adjusted model, patients in the Q2 group had a significantly lower risk of elevated SF compared to the Q1 group (OR=0.56,* p*=0.02). RCS curve analysis further confirmed the negative linear correlation between TRF and SF (non-linearity p=0.707, Figure 3), suggesting that patients with lower TRF levels are more prone to iron overload. TRF may hold significant clinical value as a predictor of iron overload.

**Table 3:** Association between TRF values and SF. Note: Crude model: unadjusted model; adjusted model 1: adjusted for age, height, weight and HbF; Adjusted model 2: adjusted for age, height, weight, HbF, RBC, RDW, Ret, HBB genotype, age at first transfusion, annual blood transfusion volume and chelation therapy. Abbreviations: TRF, transferrin; OR, odds ratio; CI, confidence interval. * represents a significant difference, *p*<0.05.

Variables	Crude model		Adjusted model 1		Adjusted model 2	
	OR (95% CI)	*p*-value	OR (95% CI)	*p*-value	OR (95% CI)	*p*-value
TRF category (median, range)						
Q1 (1.09, <1.2 g/L)	Reference		Reference		Reference	
Q2 (1.27, 1.2–1.36 g/L)	0.66 (0.44–1.01)	0.05	0.67 (0.44–1.04)	0.07	0.56 (0.34–0.92)	0.02*
Q3 (1.44, 1.36–1.53 g/L)	0.51 (0.33–0.76)	0.001*	0.51 (0.33–0.78)	0.002*	0.42 (0.26–0.69)	0.001*
Q4 (1.82, >1.53 g/L)	0.36 (0.24–0.55)	<0.001*	0.39 (0.25–0.61)	<0.001*	0.32 (0.20–0.53)	<0.001*
*p *for trend	<0.001*		<0.001*		<0.001*	

### Preoperative Hb levels and their relationship with SF

**Figure 4:**
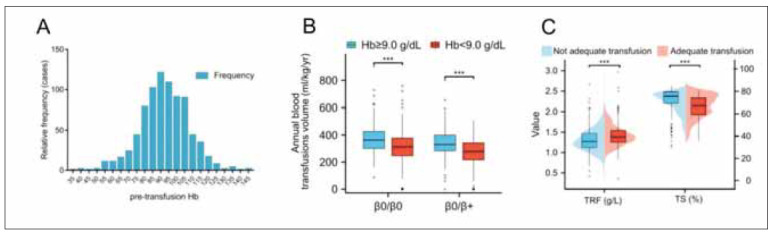
Impact of blood transfusions on TRF levels.<br>Note: (A) Frequency distribution of pre-transfusion Hb levels in the cohort of β-thalassemia patients. (B) Annual transfusion volume based on Hb levels before transfusion (≥9.0 g/dL and <9.0 g/dL) stratified by HBB genotype. (C) TRF and TS levels under different transfusion conditions. The box plots within the dot plots depict quartiles and outliers. Data are presented as mean ± standard error. ***p<0.001. Hb, haemoglobin; TRF, transferrin; TS, transferrin saturation.

According to the *Thalassemia International Federation* (TIF) guidelines, preoperative Hb levels above 9.0 g/dL are classified as »adequate transfusion. « In this study, only 58.3% of patients successfully received »adequate transfusion« to meet this standard (Figure 4A and Figure 4B). Notably, patients who achieved adequate transfusion also tended to have higher TRF levels and lower TS% (Figure 4C). However, in both the unadjusted and adjusted models, there was no significant difference in the risk of elevated SF between patients who received adequate transfusion and those who did not (Table 4).

**Table 4:** Association between pretransfusion Hb and SF. Note: Crude model: unadjusted model; adjusted model 1: adjusted for age, height, weight and HbF; Adjusted model 2: adjusted for age, height, weight, HbF, RBC, RDW, Ret, HBB genotype, age at first transfusion, chelation therapy, and annual blood transfusion volume. Abbreviations: Hb, haemoglobin; OR, odds ratio; CI, confidence interval.

Variables	Crude model		Adjusted model 1		Adjusted model 2	
	OR (95% CI)	*p*-value	OR (95% CI)	*p*-value	OR (95% CI)	*p*-value
Pretransfusion Hb category						
Pretransfusion Hb<9.0 g/dL	Reference		Reference		Reference	
Pretransfusion Hb 9.0 g/dL	0.80 (0.60–1.06)	0.13	0.81 (0.59–1.10)	0.17	0.79 (0.56–1.10)	0.17

### The protective role of HbF levels on SF

**Figure 5:**
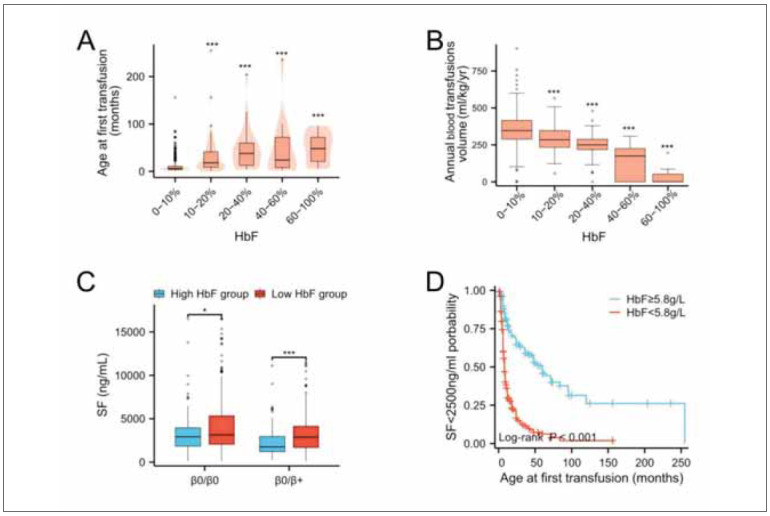
Relationship between HbF and SF in patients with severe β-thalassemia.<br>Note: HbF levels were categorised as high or low based on cut-off values determined by receiver operating characteristic (ROC) curve analysis. Kaplan-Meier curves were used to compare the probability of SF<2,500 ng/mL between high and low HbF groups, with differencesevaluated using log-rank analysis. (A) Elevated HbF levels were associated with delayed initiation of RBC transfusion. The box plots within the violin plots depict quantiles and outliers. (B) Relationship between RBC transfusions and HbF levels. (C) In the HBBgenotype group, SF levels were significantly lower in patients with high HbF. (D) Kaplan-Meier analysis shows the impact of the first transfusion age on iron overload in different HbF groups. Grey dots represent outliers in the box plot. Data are presented as mean ± SEM. *p<0.05, ***p<0.001. SF: serum ferritin; HbF: fetal haemoglobin.

**Figure 6:**
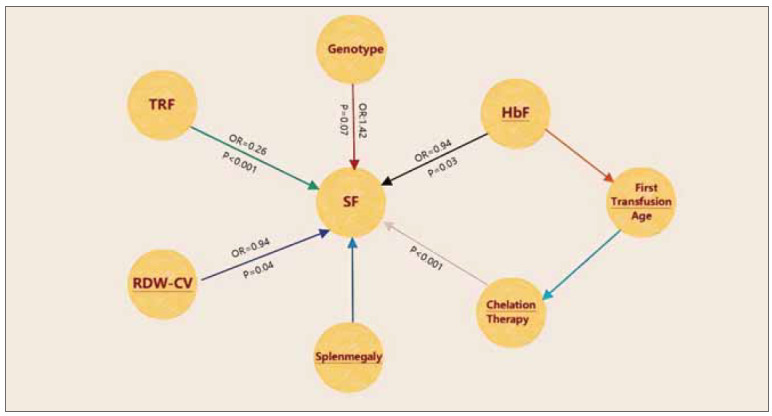
Schematic diagram of the association between TRF, SF, and other clinical variables.<br>Note: transferrin (TRF), SF, fetal haemoglobin (HbF), coefficient of variation of red cell distribution width (RDW-CV).

Finally, the study investigated the impact of HbF levels on SF. Previous research has demonstrated that HbF reactivation significantly benefits patients with β-thalassemia [18] [19] [20]. Patients with higher HbF levels typically experience a later onset of transfusion (Figure 5A) and exhibit lower transfusion dependency (Figure 5B). These patients also have significantly lower SF levels and more favourable clinical outcomes (Figure 5C and Figure 5D). These findings support the role of HbF reactivation as an effective therapeutic strategy, particularly in reducing iron overload and improving clinical prognosis. To further clarify the underlying mechanisms, a schematic illustrating the relationships between TRF, SF, and other clinical variables is presented in Figure 6.

## Discussion

### New findings in β-thalassemia

The primary findings of this study reveal a significant negative correlation between TRF levels and SF levels in patients with TDT. Multivariable-adjusted logistic regression analysis demonstrated that higher TRF levels were significantly associated with a reduced risk of elevated SF levels, further supporting the key role of TRF in regulating iron overload. This result suggests that TRF may serve as an important biomarker for managing iron overload in TDT patients. Additionally, the study found that despite frequent blood transfusions and iron chelation therapy, most TDT patients had TRF levels below the normal range. This observation implies that iron overload might suppress the synthesis or release of TRF through specific mechanisms, leading to decreased TRF levels. These findings underscore the importance of TRF in managing iron overload in TDT patients and provide new directions for investigating the mechanisms of TRF in iron metabolism.

### Role of TRF in iron metabolism regulation in TDT patients

These findings closely align with existing literature, highlighting the crucial role of TRF in maintaining iron metabolism balance, particularly under high iron overload conditions. It further reinforces the central position of TRF in iron metabolism regulation. Studies by Chen et al. [21] and Li et al. [10] indicate that TRF expression levels are closely related to iron load in the body and can influence SF levels by regulating hepatic iron storage. These studies highlight TRF’s critical role in maintaining iron metabolism balance, especially under high iron load conditions, where TRF’s role in regulating iron storage and transport becomes more important.

Unlike previous studies, this research not only validates the pivotal role of TRF in iron load regulation in TDT patients but also reveals a significant negative feedback relationship between TRF and SF levels. This negative correlation suggests that TRF may contribute to maintaining iron homeostasis by reducing excessive iron accumulation, which is important for understanding and managing iron overload in TDT patients. Additionally, TRF as a potential biomarker and therapeutic target opens new avenues for managing iron in TDT patients.

### Literature analysis and discussion

A comprehensive review of articles published from 2010 to 2024 in the PubMed database wasconducted using the keywords »In patients with Transferrin, Serum Ferritin« and »Beta-Thalassemias«. Table 5 shows these 22 studies comprehensively analyse the latest findings on clinical manifestations, diagnostic methods, treatment, and prognosis of β-thalassemia patients. The research reveals that iron overload is prevalent among β-thalassemia patients, with significant variability in ferritin levels unrelated to HFE gene mutations [22] [23]. The imbalance in ironmetabolism, combined with ineffective erythropoiesis, exacerbates health issues in these patients [4]. Chelation therapy is widely used in treatment [24] [25] [26], including deferoxamine [27] [28] [29] and vitamin C as adjunct therapy [24]. Some studies have also explored the potential benefits of natural compounds like curcumin in reducing iron load and improving liver function [30] [31]. Additionally, iron overload is linked to health issues in other systems, such as increased oxidative stress [25] [32] and depression [33]. Overall, these studies underscore the importance of managing iron overload to improve prognosis in β-thalassemia patients and highlight the need to explore new therapeutic strategies to address multisystem health impacts and enhance patients’ quality of life.

**Table 5:** A systematic review of clinical presentation, diagnosis, treatment, and prognosis in patients with beta-thalassemia from 2010 to 2024: focus on serum ferritin levels.

Journal	Authors	PMID	Year	Case Presentation	Diagnosis	Treatment	Outcome
Genet<br>Mol Res	Estev IF<br>et al. [22]	21491373	2011	Beta-thalassemia patients with HFE<br>gene mutations; analysis of serum<br>ferritin and transferrin saturation.	Beta-thalassemia	None<br>specified	Ferritin levels<br>variable regardless<br>of mutation type
Int J<br>Med Sci	Huang Y<br>et al. [4]	30745811	2019	Patients with transfusion-dependent<br>and non-transfusion-dependent<br>thalassemia; evaluation of<br>erythropoiesis and iron metabolism.	Thalassemia	None<br>specified	Iron overload and<br>erythropoiesis<br>imbalance
J Blood<br>Med	Atmakusuma<br>TD et al.<br>[26]	33883962	2019	Adult thalassemia intermedia patients;<br>correlation of serum ferritin, liver iron<br>concentration, and liver elastography.	Thalassemia<br>intermedia	Iron chelation<br>therapy	Correlation<br>between iron load<br>and liver stiffness
Transfus<br>Clin Biol	Rahmani R<br>et al. [23]	31679808	2019	Beta-thalassemia major patients;<br>investigation of HFE gene mutations<br>and serum ferritin levels.	Beta-thalassemia<br>major	Iron<br>chelation	HFE mutations<br>affect serum<br>ferritin levels
Eur J<br>Haematol	Elalfy MS<br>et al. [24]	26018112	2016	Young beta-thalassemia major<br>patients; evaluation of vitamin C as<br>adjuvant therapy to iron chelators.	Beta-thalassemia<br>major	Vitamin C and<br>iron chelation<br>therapy	Vitamin C<br>improves iron<br>chelation efficacy
Br J<br>Haematol	Zaman BA<br>et al. [34]	36535905	2023	Transfusion-dependent thalassemia<br>patients; post-transfusion changes<br>in the erythropoietin-erythroferrone-hepcidin<br>axis.	Beta-thalassemia<br>major	Blood<br>transfusions	Suppression of<br>erythropoiesis<br>after transfusion
J Blood<br>Med	Atmakusuma<br>TD et al. [35]	34526831	2021	Adult transfusion-dependent<br>beta-thalassemia patients; correlation<br>of transferrin saturation and serum<br>ferritin with bone mass density.	Beta-thalassemia	Iron<br>chelation<br>therapy	Transferrin<br>saturation<br>correlates with<br>bone density
Clin Lab	Saeidnia M<br>et al. [30]	35254032	2022	Beta-thalassemia intermedia patients;<br>effects of curcumin on iron overload.	Beta-thalassemia<br>intermedia	Curcumin	Curcumin reduces<br>iron load
Am J<br>Hematol	Elalfy MS<br>et al. [36]	37401738	2023	Infants with transfusion-dependent<br>thalassemia; evaluation of deferiprone<br>for iron shuttling to transferrin.	Transfusion-dependent<br>thalassemia	Deferiprone	Deferiprone effective<br>in preventing<br>iron overload
Cureus	Choudhary F<br>et al. [37]	36945272	2023	Beta-thalassemia major patients;<br>correlation of T-regulatory<br>cellsand iron status.	Beta-thalassemia<br>major	None<br>specified	Ferritin levels<br>correlate with<br>T-regulatory cells
Hematology	Akrawinthawong<br>K et al. [28]	21418744	2011	Transfusion-independent beta-thalassemia/<br>HbE patients; evaluation<br>of deferiprone’s effectiveness.	Beta-thalassemia/<br>HbE	Deferiprone	Deferiprone<br>reduces iron load<br>and oxidative stress
Clin<br>Pharmacokinet	Limenta LM<br>et al. [29]	21028920	2011	Beta-thalassemia patients; impact<br>of splenectomy and iron status on<br>pharmacokinetics of deferiprone.	Beta-thalassemia	Iron<br>chelation<br>therapy	Splenectomy<br>affects iron chelation<br>efficacy
Indian J<br>Hematol<br>Blood Transfus	AlFadhli S<br>et al. [38]	29075067	2017	Arab beta-thalassemia patients;<br>effects of HFE polymorphisms<br>on iron status.	Beta-thalassemia	None<br>specified	H63D polymorphism<br>alters iron<br>parameters
Mediterr<br>J Hematol<br>Infect Dis	Ragab SM<br>et al. [39]	25745546	2015	Beta-thalassemia children;<br>study of serum haptoglobin and its<br>relation to erythropoietic activity.	Beta-thalassemia	Iron<br>chelation<br>therapy	Serum haptoglobin<br>correlates with<br>erythropoietic<br>activity
Phytother<br>Res	Mohammadi E<br>et al. [31]	29806132	2018	Beta-thalassemia major patients;<br>effects of curcumin on iron<br>overload and liver function.	Beta-thalassemia<br>major	Curcumin	Curcumin<br>improves liver<br>function
Pediatr<br>Blood<br>Cancer	Inati A<br>et al. [40]	27576370	2017	Pediatric thalassemia patients;<br>comparison of phlebotomy with<br>deferasirox for treating iron overload<br>post stem cell transplantation.	Thalassemia<br>major	Phlebotomy<br>and<br>deferasirox	Both<br>treatments reduce<br>iron burden
Eur J<br>Haematol	Ghoti H<br>et al. [41]	19793250	2010	Multi-transfused sickle/beta-thalassemia<br>patients; assessment<br>of myocardial iron overload.	Sickle/beta-thalassemia	Iron<br>chelation<br>therapy	No evidence of<br>myocardial iron<br>overload

### Clinical significance

This study demonstrates that dynamic changes in TRF levels are more sensitive to iron load increases in the early stages and can serve as an important biomarker for monitoring iron metabolism imbalance. Under iron overload conditions, changes in TRF often precede those in storage markers like SF, making TRF highly promising for early-stage iron load assessment.

In contrast, SF only rises significantly once iron overload reaches a certain threshold. While MRI can accurately assess tissue iron deposition, its high cost and complexity limit its application in early diagnosis. Therefore, incorporating TRF into routine monitoring could enable clinicians to implement timely interventions in the early stages of iron overload, thus more effectively preventing iron accumulation and associated complications.

The 2021 Guidelines for the Treatment of Thalassemia recommend assessing iron overload using SF levels and cardiac and hepatic MRI. However, TRF has not yet been included as a routine monitoring parameter. Dynamic TRF monitoring can aid clinicians in accurately assessing iron load and guide individualised chelation therapy. For high-risk TDT patients, regular TRF monitoring allows for timely identification of iron overload risk, enabling adjustments to chelation dosage and frequency tailored to the patient’s specific needs. This approach optimises treatment and reduces the risk of complications such as cardiac, endocrine, and hepatic dysfunction. For patients with elevated TRF levels and complex iron load management, closer monitoring and adjustments to transfusion strategies and chelation therapy may be necessary to ensure long-term treatment balance and mitigate the risk of complications.

Additionally, studies indicate that increasing fetal haemoglobin (HbF) levels can reduce transfusiondemand, thereby decreasing iron load. Increased HbF can improve haemoglobin levels in patients,reduce dependence on exogenous iron, and further enhance the value of TRF monitoring.

In conclusion, this study supports TRF as a crucial monitoring tool for individualised treatment in TDT patients. Combined with HbF monitoring, TRF enables precise prediction and prevention of iron overload-related complications, enhancing patients’ quality of life and clinical outcomes. This integrated strategy not only advances new directions in iron metabolism management but also lays the foundation for developing personalised treatment plans with broad clinical applications.

### Limitations and future research directions

The main limitation of this study lies in its cross-sectional design, which restricts causal inference between TRF and SF. Additionally, adherence to chelation therapy was not fully assessed, potentially affecting the accuracy of the results. Future studies should adopt a longitudinal design to track dynamic changes in TRF and SF and further explore their causal relationship.

At the same time, genome-wide association studies (GWAS) and other methods can analyse genetic polymorphisms affecting TRF expression and function, revealing regulatory mechanisms and providing new insights for individualised treatment in TDT patients.

This study found that elevated TRF levels are often accompanied by a decline in SF, suggesting apossible dose-dependent relationship between them. However, the precise causal relationship remains unclear. To further understand whether TRF directly regulates systemic iron load, future studies should utilise animal models or clinical longitudinal studies to investigate TRF’s role in iron metabolism. For instance, in thalassemia mouse models, modulating TRF expression and observing its effects on iron load and SF levels may provide more direct evidence supporting TRF’s role in managing iron overload.

Iron metabolism is a complex network of regulatory interactions involving transferrin (TRF), ferritin,hepcidin, ferroportin, and divalent metal transporter 1 (DMT1). Future research should focus on the interplay between TRF and these proteins under different iron overload conditions, providing a broader perspective on TRF’s functional role in iron metabolism.

Longitudinal clinical studies can help evaluate the dynamic relationship between TRF and SF, clarifying their temporal sequence and verifying TRF’s potential regulatory role in iron metabolism. If increasing TRF is proven to directly reduce iron overload, it could serve as a therapeutic target, enabling more effective personalised treatment strategies for patients with transfusion-dependent thalassemia (TDT).

Future treatment approaches should further advance toward precision and individualised therapy. By considering patients’ genotypes and iron metabolism profiles, clinicians can develop more targeted strategies to manage iron overload and reduce related complications. Personalised treatment plans may include gene therapy, targeted drugs, and novel combination therapies, particularly optimised for patients with specific genetic profiles.

## Conclusion

This study investigates the relationship between TRF and SF in patients with TDT. Genotypic and phenotypic analyses were conducted on 817 patients, revealing a significant inverse linear relationship between elevated TRF levels and reduced SF levels. Logistic regression and RCS analyses further confirmed this dose-response relationship, suggesting that TRF may be protective in regulating iron overload. These findings indicate that TRF is not only associated with iron metabolism but may also serve as an important biomarker for managing iron overload and preventing its related complications.

This study identifies TRF as a novel biomarker for managing iron overload in patients with β-thalassemia. Monitoring and regulating TRF levels may be crucial in reducing iron burden and preventing complications caused by iron overload, such as heart disease and liver disorders. Clinically, assessing TRF levels could enable physicians to predict the risk of iron accumulation more accurately, leading to the development of more personalised and effective treatment strategies. Moreover, these findings provide a theoretical foundation for future therapeutic innovations.

Although this study provides valuable insights into the relationship between TRF and SF, several limitations should be acknowledged. First, it is difficult to establish a causal relationship as a cross-sectional study. Second, the sample was limited to TDT patients with specific genotypes, which may affect the generalizability of the findings. Additionally, the study did not account for all variables influencing iron metabolism, such as dietary habits and other underlying health conditions, which may have impacted the results.

Future research should consider adopting a longitudinal design to more accurately determine the causal relationship between TRF and SF. Including a broader population and a wider range of variables will also help validate the universality and robustness of these findings. Moreover, exploring the role of TRF in other types of iron overload disorders presents a promising avenue for future investigation. Based on these findings, developing TRF-targeted therapies could offer a novel strategy for treating iron overload diseases, which warrants further exploration and validation in future clinical trials.

## Dodatak

### Acknowledgements

None.

### Ethics statement

This study was approved by the ethics committee of Nanfang Hospital, Southern Medical University (approval no. NFEC-2019-039), and the ethics committees at each local hospital. All subjects and/or their guardians provided signed informed consent for participation. This study adhered to the Declaration of Helsinki.

### Author contributions

YL designed the study. YL and YH analysed the data. YL and YY drafted the manuscript. BL conducted the experiments. XZ, BH, LL, YH, KH, JL, XF, XL, ZL, WZ, LY, DT, and TZ were responsible for patient recruitment. XX supervised the research. All authors reviewed and approved the final manuscript.

### Funding

This work was supported by grants from the National Key Research and Development Program of China (grant nos.2018YFA0507800 and 2018YFA0507803) and the Open Project of BGI-Shenzhen, Shenzhen 518000, China (BGIRSZ20200008).

### Data availability

All data can be provided as needed.

### Conflict of interest statement

All the authors declare that they have no conflict of interest in this work.

### List of abbreviations

CIs, confidence intervals;<br>DMT1, divalent metal transporter 1;<br>GWAS, genome-wide association studies;<br>Hb, haemoglobin;<br>HbF, fetal haemoglobin;<br>HPFH, hereditary persistence of fetal haemoglobin;<br>NGS, next-generation sequencing;<br>NTDT, non-transfusion-dependent beta-thalassemia;<br>ORs, odds ratios;<br>PCR, polymerase chain reaction;<br>RBC, red blood cell;<br>RCS, restricted cubic spline;<br>RDB, reverse dot blot;<br>RDW, red cell distribution width;<br>RDW-CV, coefficient of variation of red cell distribution width;<br>ROC, receiver operating characteristic;<br>SF, serum ferritin;<br>SI, serum iron;<br>TDT, transfusion-dependent beta-thalassemia;<br>TIBC, total iron-binding capacity;<br>TIF, Thalassemia International Federation;<br>TRF, transferrin;<br>TS, transferrin saturation;<br>β-thalassemia, beta-thalassemia.
